# Supplementary material for: Absence of reliable physiological signature of illusory body ownership revealed by fine-grained autonomic measurement during the rubber hand illusion
Source: PLoS One. 2021 Apr 1;16(4):e0237282. doi: 10.1371/journal.pone.0237282 (PMC8016256; doi:10.1371/journal.pone.0237282)
Supplement: S2 File — (DOCX) [file pone.0237282.s002.docx]

**Absence of reliable physiological signature of illusory body ownership revealed by fine-grained autonomic measurement during the rubber hand illusion**

Hugo D Critchley^1,2,3*^, Vanessa Botan^1,2^, Jamie Ward^1,^**^2,^**

**SUPPLEMENTARY MATERIAL:**

**A: Additional analyses**

1. **Non-parametric test results**
2. **Rubber hand illusion subjective ratings**

Non-parametric Wilcoxon tests were run comparing subjective ratings for each of the subscales between synchronous and asynchronous conditions indicating the following results for ownership (Z=-4.810, p<0.001); location (Z=-4.452, p<0.001); agency (Z=-3.977, p<0.001).

1. **Rubber hand illusion subjective ratings low versus high interoception**

Non-parametric Mann-Whitney U tests were run to assess differences between low and high interoceptive accuracy groups for each of the conditions and subscales. The results obtained were: synchronous ownership (Z=-1.035, p=0.301); synchronous location (Z=-0.451, p=0.652); synchronous agency (Z=-0.226, p=0.821); asynchronous ownership (Z=-1.009, p=0.313); asynchronous location (Z=-0.417, p=0.986); asynchronous agency (Z=-0.036, p=0.972).

1. Skin sympathetic nerve activity (SKNA)

Non-parametric Wilcoxon tests were run comparing average skin sympathetic nerve activity (aSKNA) for each time-window between synchronous and asynchronous conditions indicating the following results:

30s (Z=-1.237, p=0.216); 60s (Z=-0.312, p=0.755); 90s (Z=-0.118, p=0.906); 120s (Z=-1.769, p=0.077).

Non-parametric Mann-Whitney U tests were run to assess differences in aSKNA between participants who got the illusion and participants who did not get the illusion for each time-window providing the following results: synchronous 30s (Z=-0.417, p=0.677); synchronous 60s (Z=-0.547, p=0.585); synchronous 90s (Z=-0.899, p=0.368); synchronous 120s (Z=-0.016, p=0.987); asynchronous 30s (Z=-0.193, p=0.847); asynchronous 60s (Z=-0.048, p=0.962); asynchronous 90s (Z=-0.548, p=0.604); asynchronous 120s (Z=0.809, p=0.814).

**NB:**

The SKNA and SCR data were not normally distributed, not even after logarithmic transformation was applied. There is no Bayesian statistical analysis available for not-normally distributed data. As such, non-parametric test results were made available in the appendix. Also, there is no Bayesian analysis available for repeated measures ANOVAs (within the statistical platform used here: SPSS) so the four dependent measures were compared separately. Lastly, it might be better not to include the results for the asynchronous condition between participants who got the illusion and those who didn’t. The discriminant analysis was based on the fact that some people got the illusion in the synchronous condition and not in the asynchronous one.

**B: Data files**
